# An efficient photograph-based quantitative method for assessing castrating trematode parasites in bivalve molluscs

**DOI:** 10.1017/S0031182020001213

**Published:** 2020-10

**Authors:** Joshua I. Brian, David C. Aldridge

**Affiliations:** Aquatic Ecology Group, The David Attenborough Building, Department of Zoology, University of Cambridge, Cambridge CB2 3QZ, UK

**Keywords:** *Anodonta*, bivalve, castration, cercariae, gonad, sporocyst

## Abstract

Parasitic castration of bivalves by trematodes is common, and may significantly reduce the reproductive capacity of ecologically important species. Understanding the intensity of infection is desirable, as it can indicate the time that has passed since infection, and influence the host's physiological and reproductive response. In addition, it is useful to know the developmental stage of the trematode, to understand trematode population trends and reproductive success. However, most existing methods (e.g. visually estimating the degree of infection) to assess intensity are approximate only and not reproducible. Here, we present a method to accurately quantify the percentage of bivalve gonad filled with digenean trematode tissue, based on small squashes of gonad tissue rapidly photographed under light microscopy. A maximum of 15 photographs is required to determine the percentage of the whole gonad occupied by trematodes with a minimum of 90% confidence, with smaller mussels requiring fewer. In addition, the stage of trematode infection can be assessed because full sporocysts, spent sporocysts and free cercariae are clearly distinguishable. Although variation exists in the distribution of trematodes in gonad tissue, and thus in the estimate of percentage of the gonad filled with trematodes, this method represents a marked improvement on current coarse assessments of infection which typically focus on binary presence/absence measures. This technique can be used to facilitate a more sophisticated understanding of host–parasite interactions in bivalves, and can inform the conservation and reproductive biology of environmentally crucial species.

## Introduction

Parasites face a classic trade-off between maximizing host exploitation and maintaining host longevity. A proposed evolutionary solution is castration of the host, which allows for complete redirection of host reproductive energy towards the parasite, while facilitating continued host survival (Baudoin, 1975; Lafferty and Kuris, 2009). Castration has a wide range of potential consequences on the host, including timing and success of reproduction, changes in distribution and overall energy allocation dynamics (Lafferty and Kuris, 2009), which can influence population-level reproductive output (Fredensborg *et al*., 2005). The types of consequences will depend on a range of factors including prevalence (what proportion of the population is infected), intensity (how many parasites, or how much parasite tissue, is present in a single individual) and infection biases such as age, sex and size. Parasitic castration is evident throughout the animal kingdom, but is particularly common in fish (e.g. Jobling and Tyler, 2003) and molluscs (e.g. Averbuj and Cremonte, 2010; Choubisa and Sheikh, 2013; Yee-Duarte *et al*., 2017).

The castration of molluscs, and in particular bivalves, is valuable and important to study for three reasons. First, bivalve population dynamics are of specific concern given they often fulfil important ecosystem engineering functions through filter-feeding, burrowing and providing a hard substrate to increase niche complexity (Sousa *et al*., 2009; Lopes-Lima *et al*., 2017). For example, through their filtering they can modify nearby water chemistry (Ninokawa *et al*., 2020), act as important nutrient cyclers (Atkinson *et al*., 2010) and form nutrient hotspots (Atkinson and Vaughn, 2015). As a result, their presence is associated with increased species richness in both marine and freshwater ecosystems (Aldridge *et al*., 2007; Borthagaray and Carranza, 2007; Chowdhury *et al*., 2016). Second, bivalves include some of the most globally imperilled taxa (Bogan, 1993; Smith *et al*., 2006; Lopes-Lima *et al*., 2018) and effective conservation programmes may benefit from understanding drivers of reduced fecundity. Third, bivalves are often found at high density, vary in possible resource availability to parasites in predictable and easily measurable ways (e.g. size, sex and gravidity) and are amenable to simple manipulation. These qualities make them a potential model system to study the individual, population-level and evolutionary effects of parasitic castration.

Digenean trematodes are common castrating parasites of bivalves, which utilize them as a first intermediate host (see Brian and Aldridge, 2019). Digeneans fill the gonad with asexually-reproducing sporocysts, rediae or both, which produce cercariae that are eventually released to infect the next host. This continued asexual growth eventually leads to castration of the bivalve host. These infections are chronic, and can last for life (Taskinen *et al*., 1997). Digeneans may also infect bivalves as a second intermediate host in the form of metacercariae. However, understanding the effects of digenean castration on bivalves is currently hindered by an inability to reliably quantify the level of infection, as the asexual branching growth of sporocysts means there are no specific ‘individuals’ that can be counted.

Evidence suggests that to understand the population-level effects of infection, quantitative data are required. For example, previous research indicates that being castrated (or not) and being infected (or not) should not be treated as binary variables. Taskinen and Valtonen (1995) demonstrated that 18.5% of mussels could still reproduce when infected with sporocyst tissue (though the level of infection for each mussel was not recorded). This suggests that mussels can still reproduce at some level of infection, and that sporocysts need to fill a certain proportion of the gonad before the bivalve is castrated. They further demonstrated that an increased quantity of sporocysts led to reduced number of host eggs being produced by females. Other studies have also suggested that an increased volume of trematode tissue can lead to more cercariae being produced (e.g. Hay *et al*., 2005; Thieltges *et al*., 2008), showing that infection intensity may have consequences for both host and parasite populations. However, most studies examining castrating parasites in bivalves use a small portion of the gonad, and either record infection status as a binary yes/no (e.g. Valderrama *et al*., 2004; Baudrimont *et al*., 2006; Zieritz and Aldridge, 2011; Marszewska and Cichy, 2015), or provide granular assessments of infection such as ‘low’ or ‘high’ (e.g. Taskinen *et al*., 1994, 1997; Yanovich, 2015). Binary records of infection may not be enough to capture the nuances of host–parasite dynamics, whereas the granular assessments of intensity are highly subjective and not reproducible, hampering efforts to compare population impacts between studies and locations. A better metric would potentially be a measure of intensity of infection, such as the percentage of the gonad filled with trematode tissue. Such an explicit quantitative measure is likely to correlate directly with the amount of tissue available for mussel and parasite reproduction. In addition, more comprehensive methods such as histology are time-consuming, require a high level of skill and may still lack objectivity unless a clear quantitative procedure is followed.

In this paper, we present a rapid photograph-based method to quantify the infection level within the gonad of a mollusc. It provides a reproducible measure of the percentage of the gonad filled, and can capture within-host trematode dynamics, by distinguishing between full sporocysts, spent sporocysts and free cercariae. Although this technique is exemplified with unionid mussels, we see it as applicable to all marine and freshwater bivalves.

## Methods

### Study site and collection

The duck mussel *Anodonta anatina* (Linnaeus 1758) is a common unionid with a pan-European distribution (Lopes-Lima *et al*., 2017). We collected samples of this mussel on a monthly basis between January and September from the Old West River at Stretham (52.3343°N, 0.2243°E), part of the River Great Ouse system (UK). Exploratory dissections had revealed a proportion of these mussels to be infected with the digenean trematode *Rhipidocotyle campanula* (Dujardin 1845); this trematode produces cercariae from asexual sporocysts. We collected mussels by hand from near the bank, and transported back to the laboratory in 10 L buckets filled with river water. In the laboratory, we held mussels at 8°C under aeration, for a maximum of 72 h before assessments of trematode infection. Prior to assessment, we rinsed mussels under cold fresh water while holding the valves gently shut to remove any organisms on the shells, and measured maximum length to the nearest 0.5 mm with a Vernier callipers.

### Quantification of trematode infection

We sacrificed and dissected mussels by inserting a scalpel between the valves and slicing the posterior and anterior adductor muscles. We gently removed the visceral mass by cutting the connective tissue at each end, and sliced it open using a single cut of a scalpel at the posterior end.

To confirm that a subsample of the gonad will approximate the true percentage of the gonad filled with trematode tissue, we quantified the entire gonad of 10 mussels of variable size. We repeatedly removed ~40 mg samples of gonad tissue with tweezers, and pressed individual samples gently between two glass microscope slides to create a squash ~10 mm in diameter. Although this method describes the technique for bivalves, this could equally be applied to the isolated gonad of any mollusc. We repeated this until there was no more gonad tissue left in the visceral mass. We inspected each glass microscope slide thoroughly at 40× magnification using a GXM-L3200 compound microscope. Where present, we captured photos of trematode material in .tif format using a HiChrome-S camera attached to the microscope and software GX Capture 8.5 (loaded on a standard Windows PC). Each photograph captured an area of 1630 × 917 *μ*m. We took three photographs for each squash (i.e. a single 40 mg sample), resulting in *n* photographs (*n* ranged between 18 and 48, depending on the size of the mussel).

**Fig. 1.**
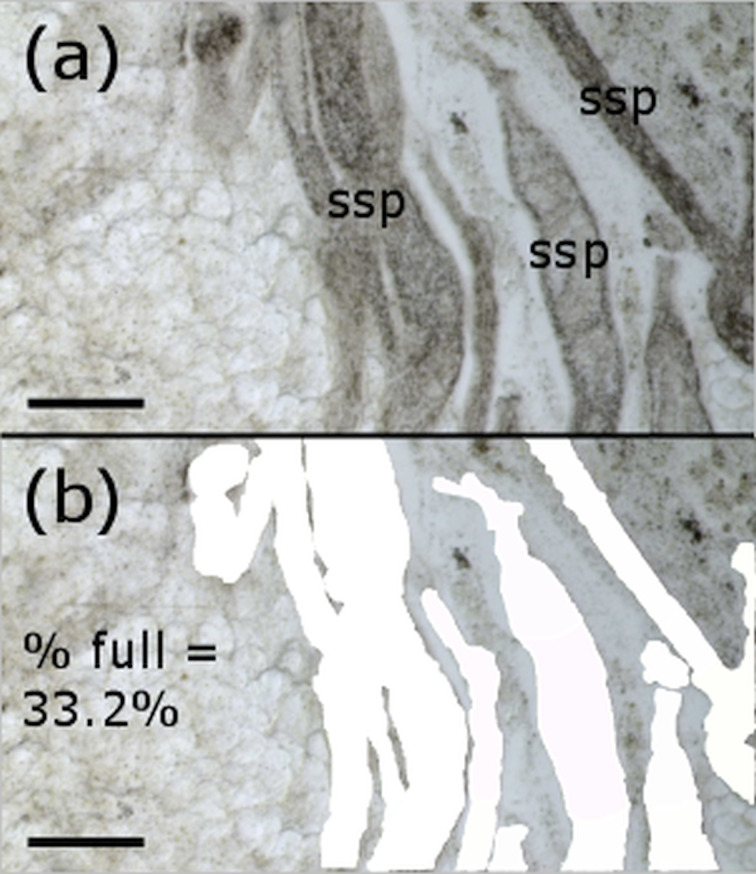
Tracing and measuring procedure for a single photograph. Scale bars = 250 *μ*m. (a) An example photo, showing spent sporocysts (ssp). (b) The traced sporocysts and their cumulative area (white), with the corresponding percentage of picture filled with sporocyst.

Based on the extensive dissections and subsampling of the 10 mussels we concluded that assessing 12 photographs from an individual mussel generally provided reliable data on infestation levels (see Sections ‘Results’ and ‘Discussion’). Therefore, for all other mussels, we removed four replicate samples of gonad tissue with tweezers (~40 mg each), and took three photos per sample from a random point in the squash. We took each sample from a different area of the gonad, namely the dorsal and ventral areas on each side of the slice. We loaded each .tif image into ImageJ 2.0.0, and carefully traced around the digenean tissue (sporocysts and cercariae) using the ‘Freehand selection’ tracing tool. Using the ‘Measure’ function in ImageJ, we calculated the total area of each trace, and hence the percentage of the photo that contained trematode material (Fig. 1). We averaged this value over all 12 replicate photos, to give the mean percentage of infected gonad for each mussel. This procedure was performed for all mussels to confirm the method's appropriateness throughout the year, when the proportion of sporocysts and cercariae may change.

### Statistical analysis

All statistical analyses were executed in R v3.5.1 (R Core Team, 2018). To confirm that a subsample of the gonad approximated the true percentage of the gonad filled with trematode tissue, we analysed the 10 mussels that had their entire gonad quantified (using *n* photographs) in the following fashion. We calculated the percentage of the gonad filled with trematode for each photo, averaged all photos to the give the true mean percentage of gonad filled for each mussel and then calculated an associated 99% confidence interval. Subsequently for each mussel, we took a random subsample of photographs without replacement (beginning with two photographs, then three, up to *n* photographs), and calculated the mean percentage of photo filled with trematode. We repeated this procedure 1000 times for each number of photographs, and calculated the proportion of those replicates which had the estimated mean falling inside the 99% confidence interval of the true mean. To understand how this was affected by mussel size, we compared the number of photographs required to have 90% of replicates approximating the true mean with the length of the mussel using linear regression. We checked and confirmed assumptions of linearity, normality and homoscedasticity of residuals using *Q*–*Q* and residuals *vs* predicted values plots.

## Results

**Fig. 2.**
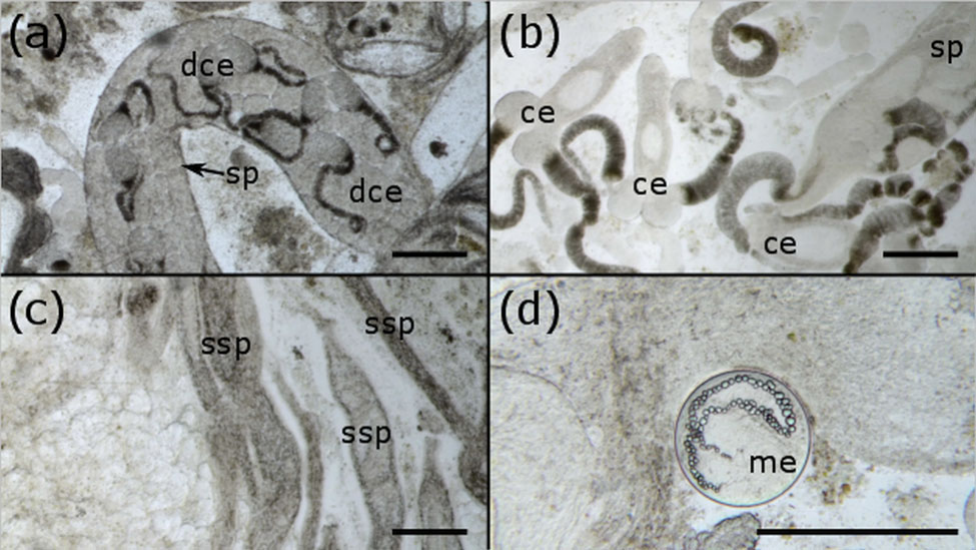
Different developmental stages of digenean trematodes occupying *A. anatina*, as captured by photography. All scale bars = 250 *μ*m. (a) Full sporocyst (sp) with developing cercariae (dce) inside. (b) Free cercariae (ce), ready to be released and infect a second intermediate host. (c) ‘Spent’ sporocysts (ssp), having released their cercariae. (d) Metacercariae (me) of echinostomatid trematodes were also occasionally observed, utilizing the mussel as a second intermediate host. Small bubbles within the metacercaria are excretory vacuoles.

Overall, trematode infections of varying intensity were observed in 72 mussels (17.1% of all mussels examined), based on the inspections of the squashes from the gonad dissections (min: 0.2% of gonad filled with trematode tissue; max: 78.1%). Photographs of these squashes produced high-quality images throughout the year that were used to calculate the percentage area of the gonad filled with trematode tissue, and accurately characterize the developmental stages of trematode infection (Fig. 2).

**Fig. 3.**
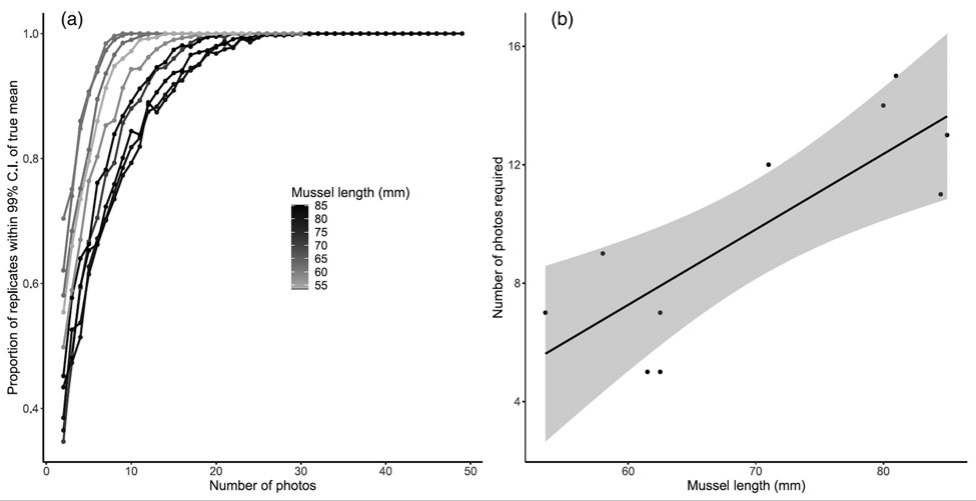
Accuracy of the method for mussels of variable size. (a) Relationship between the number of photographs taken (each point represents 1000 random samples of that number of photographs, from the pool of all possible photographs), and the proportion of those 1000 replicates where the estimated mean trematode percentage fell within the 99% confidence interval of the true mean trematode percentage. Each mussel (*n* = 10) is plotted separately, and shaded according to its length in mm. (b) Linear regression between the length of the mussel, and the number of photos required to have 90% of the replicates approximating the true mean. Shading denotes the 95% confidence interval of the fitted line.

The amount of trematode tissue present varied between photographs, and shows the utility of replication of photographs for a single mussel. Additionally, as the number of photographs increased, success at estimating the true mean increased rapidly (Fig. 3). Figure 3a shows that the number of photographs required to reliably estimate the true mean is dependent on the size of the mussel, with smaller mussels approaching the true mean faster. This trend is confirmed by Fig. 3b, which demonstrates the significance of this relationship (*R*^2^ = 0.67, *P* = 0.004). However, all mussels had at least 90% of replications approximating the true mean after 15 photographs (Fig. 3b), and Fig. 3 suggests that for the mean mussel size in this study (65 mm), 10 photographs are appropriate to estimate the true mean.

## Discussion

### Recommendations

A gonad squash technique has been described for the quantitative assessment of trematode infection in bivalve gonads. This method is fast and simple to use, and provides clear evidence for the presence of trematode infection, in addition to the developmental stage of the trematodes (Fig. 2). Furthermore, we have demonstrated that small sub-samples of the gonad rapidly capture the pattern in the whole gonad (Fig. 3). We therefore recommend that:

1. the number of photographs taken is determined by the size of the bivalve;
2. the developmental stage of the trematode is reported in addition to the infection intensity;
3. the photographs are stored in an appropriate digital repository;
4. future studies of parasitic castration in bivalves utilize this method to quantitatively assess infection.

The rationale behind these recommendations is briefly discussed below.

### Assessing method success

There is a high level of variation evident between photographs within a single mussel. However, variation is not unexpected, given the previously observed uneven distribution of trematode infection within bivalve gonads (Taskinen *et al*., 1997). However, Fig. 3 shows that for an average mussel 65 mm long, 10 photographs will reliably estimate the true mean percentage of the gonad filled with trematode tissue. To balance time considerations against predictive power, we have recommended basing the number of photographs on the size of the bivalve, but we note that for all except for the largest mussels, a conservative 12 photographs may be an appropriate number to analyse, as this linear relationship could vary between species. Although there may still be variation present around estimates, the presented method represents a significant improvement on current quantitative efforts, where intensity is generally classed into a maximum of three categories (e.g. ‘low’, ‘medium’ and ‘high’). This technique has an additional advantage in that it is reproducible, assessable by other researchers (e.g. photos can be made publicly available for inspection) and therefore can be used to compare infection prevalence and intensity across multiple studies executed by a diverse range of researchers.

We consider our method to stand up favourably to alternative procedures. Histological examination of gonadal cross-sections has been used to determine the intensity of infection in bivalve-trematode systems (e.g. Lajtner *et al*., 2008; Ceuta and Boehs, 2012). However, we see cross-sections as problematic. As histology uses very thin cross-sections, the resulting image for analysis is reliant on the orientation of the sporocyst in the plane that the section was taken. A transverse section of sporocyst would give a very low area, whereas a longitudinal section would give a very large area, even though both represent a single sporocyst. Our technique, which takes 3D samples and then gently compresses them, provides a more natural approximation of how much of the gonad is represented by trematode tissue. In addition, it is a lot less labour-intensive than histology, which requires setting of material in paraffin, sectioning and staining (Laruelle *et al*., 2002). In contrast, our technique can produce images within 5 min of the mussel being dissected, ready to be analysed at the researcher's convenience, thus increasing efficiency. Estimating the proportion of mollusc biomass contributed by trematodes has also been used to quantify infection, by separating host and parasite tissue (e.g. Preston *et al*., 2013). However, it can be very difficult to separate host and parasite tissue (Kuris *et al*., 2008), leading to estimates of parasite mass based on cross-sections of host tissue (e.g. Hechinger *et al*., 2008), a method that has the potential to result in similar errors to those associated with histology. In addition, weighing such light quantities can be a long and complex procedure (e.g. see Lambden and Johnson, 2013). Therefore, the method presented here represents a simpler procedure with at least as much accuracy. There is the potential for the analysis of photographs to be further automated, through the training of computer algorithms to recognize sporocyst tissue. Automation was met with difficulties in the current study (see Fig. S1 and associated discussion in Supplementary material), but provides a potential avenue for future development.

### Utility of the technique in improving parasite studies

Future studies investigating the effect of parasitism on bivalves could benefit from the method presented in this paper. Currently, when the effect of parasitism on a quantitative variable (e.g. gene expression, phenoloxidase activity or host fecundity) are explored, parasitic state is often defined simply as ‘parasitized’ or ‘unparasitized’ (e.g. Valderrama *et al*., 2004; Baudrimont *et al*., 2006; Magalhães *et al*., 2017). This approach does not accord with evolutionary theory which predicts that at initial infection and low parasite intensity, hosts may invest heavily in reproduction and defence, whereas at high intensity they will direct resources elsewhere (Hurd, 2001; Lafferty and Kuris, 2009). In short, a bivalve that is uninfected and a bivalve that has 5% of their gonad filled are likely to be more physiologically similar compared to two infected bivalves that have 5 and 95% of their gonad filled, respectively. This point is also supported by experimental data and field observations (Sorensen and Minchella, 2001; Munoz *et al*., 2006). However, studies that explicitly quantify the effect of infection intensity on host or parasite dynamics are rare, and methods like the one presented in this paper should be used wherever possible. Nonetheless, it is erroneous to treat parasitism as a binary variable, as infection intensity instead represents a continuum, with variable host responses predicted along it (Kabat, 1986). In general, increased parasitic intensity may lead to increased host mortality (Mouritsen and Poulin, 2002), making its characterization crucial to understand the outcomes of parasitism for both individual hosts and host populations. The technique described in this paper provides a simple way of assessing the level of infection, not just whether it is present or not, and therefore facilitates a more nuanced characterization of host responses to parasite infection. In addition, the clear ability of the method to distinguish between developmental stages of the trematode should be taken advantage of to understand parasite development both within and between host individuals.

In summary, the method presented facilitates the rapid and realistic quantification of trematode infection in bivalve gonads. Given the important ecological role of bivalves, their conservation status and their potential as a model host–parasite system, we strongly advocate for the use of this technique to enhance biological understanding in future observational or experimental studies.

## Supplementary material

For supplementary material accompanying this paper visit https://doi.org/10.1017/S0031182020001213. click here to view supplementary material
