# Supplementary material for: An efficient photograph-based quantitative method for assessing castrating trematode parasites in bivalve molluscs
Source: Parasitology. 2020 Jul 30;147(12):1375–80. doi: 10.1017/S0031182020001213 (PMC7477367; doi:10.1017/S0031182020001213)
Supplement: Supplementary file 1 [file S0031182020001213sup001.docx]

**A rapid photograph-based quantitative method for assessing castrating trematode parasites in bivalve molluscs**

**Joshua I. Brian, David C. Aldridge**

**Supplementary Material**


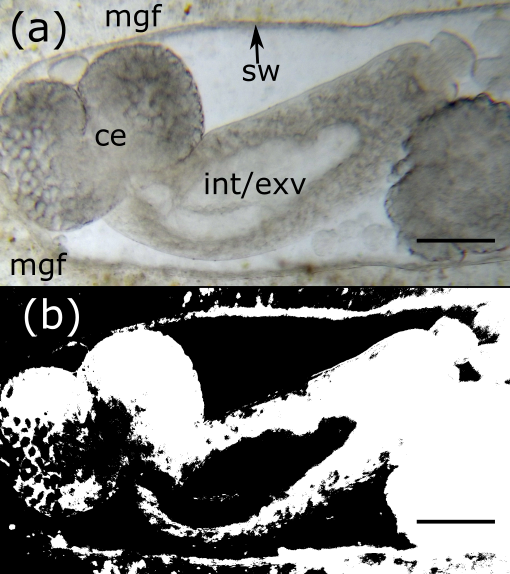


Figure S1: Demonstration of an issue with potential computer automation of sporocyst/cercariae recognition, using a close-up of the end of a sporocyst with a developing cercaria inside. Scale bars 250 μM. (a) The developing cercaria (ce) inside the sporocyst wall (sw), with the sporocyst residing in mussel gonadal fluid (mgf). Note the light-coloured developing intestine (int) and excretory vesicle (exv) inside the cercaria. (b) The results of a binary threshold classification of (a) in ImageJ.

Figure S1 shows that automation of the tracing and percentage filled procedure may be difficult. Everything inside the sporocyst wall (Fig. S1a) should be included as trematode tissue; however, the binary threshold mask (Fig. S1b) cannot distinguish between mussel gonadal fluid, the interior of the sporocyst, and intestine/excretory vesicle of the cercaria. Multiple different thresholding algorithms yielded similar results. While it is undoubtedly possible for automation of this procedure, it was beyond the remit of the current study.
